# Microbial Diversity in the Era of Omic Technologies

**DOI:** 10.1155/2013/958719

**Published:** 2013-10-24

**Authors:** Sofia Nikolaki, George Tsiamis

**Affiliations:** Department of Environmental and Natural Resources Management, University of Patras, 2 Seferi Street, 30100 Agrinio, Greece

## Abstract

Human life and activity depends on microorganisms, as they are responsible for providing basic elements of life. Although microbes have such a key role in sustaining basic functions for all living organisms, very little is known about their biology since only a small fraction (average 1%) can be cultured under laboratory conditions. This is even more evident when considering that >88% of all bacterial isolates belong to four bacterial phyla, the *Proteobacteria*, *Firmicutes*, *Actinobacteria*, and *Bacteroidetes*. Advanced technologies, developed in the last years, promise to revolutionise the way that we characterize, identify, and study microbial communities. In this review, we present the most advanced tools that microbial ecologists can use for the study of microbial communities. Innovative microbial ecological DNA microarrays such as PhyloChip and GeoChip that have been developed for investigating the composition and function of microbial communities are presented, along with an overview of the next generation sequencing technologies. Finally, the Single Cell Genomics approach, which can be used for obtaining genomes from uncultured phyla, is outlined. This tool enables the amplification and sequencing of DNA from single cells obtained directly from environmental samples and is promising to revolutionise microbiology.

## 1. Introduction

Microbes are essential for every part of the human life on Earth as they are responsible for converting the key elements of life—carbon, nitrogen, oxygen, and sulfur—into forms accessible to all other living things [1]. Even more interestingly, the majority of the photosynthetic capacity of the planet does not depend on plants but on microbes [2]. Microbial communities are closely associated with plants and animals making necessary nutrients, metals, and vitamins available to their hosts. For humans, the billions of gut microbes assist us to digest food, break down toxins, and fight off pathogens [2]. Humanity not only depends on microbes for nutritional and health reasons but also for cleaning up pollutants in the environment, such as oil and chemical spills [2]. Amazingly, these activities are not carried out by individual microbes but by microbial communities that can adapt and excel even under extreme environmental changes. These communities can live under extreme conditions, at pH level, pressure, and temperatures, in which no other organism can survive. This has been achieved through numerous strategies that have been developed by microbes for survival. Their genomes contain a vast array of biochemical transformations, and the microbial cells have accumulated DNA changes over a period of billion years of environmental change and evolution [3].

Human civilization has been greatly improved by the development of numerous technologies that have their source in microbes. For instance, they are being used to produce a vast array of antibiotics and drugs for clinical use, to remediate pollutants in soil and water, to produce biofuels, to enhance and protect agricultural crops, and to ferment human foods and even they are used as markers for the detection of diseases [2, 3].

Comparative analysis of ribosomal RNA (rRNA) sequences implied that all of the cellular life belonged to one of the three domains, namely, Bacteria, Archaea, and Eukarya [4]. This enabled the definition of the major lineages (phyla or divisions) within the three primary domains [5]. Microbes are the most diverse group on Earth on the basis of phylogeny and functionality, occupying every conceivable niche. The vast majority of these organisms are characterized through culture-independent molecular surveys using conserved marker genes like the small subunit ribosomal RNA or more recently the shotgun sequencing (metagenomics) [6, 7]. Although microbes are such an important group, the characterization, identification, and quantification remain an immense challenge even with the conventional molecular tools [8, 9].

The most important revolution in microbial ecology was the use of DNA sequencing in phylogenetic studies and the application of this technology to uncultured organisms in the 1980s and the 1990s. This transformed microbiology revealed that the prokaryotic diversity was vastly underestimated with the current classical cultivation-based techniques [4, 6, 7, 10, 11]. In the last ten years, metagenomic projects have been combined with next generation sequencing (NGS) technologies. This has boosted microbial ecology forward in a very fast pace [1, 12, 13]. Current NGS technologies provide a throughput, which is at least 100 times that of classical Sanger sequencing, and the technologies are quickly improving [14–16]. This makes NGS one of the hottest topics in biological sciences. With the assistance of the newly developed discipline of metagenomics and the high-throughput sequencing technologies, scientists can now unravel the mysteries of the life of the still uncultured microorganisms [6, 17]. The use of metagenomic approaches led to the discovery of a large array of new genes and enabled the genome sequence of various uncultured microbes [18–20]. This is true for low to medium complex ecosystems. In highly diverse environments, metagenomic approaches have not been so successful since assembly is extremely challenging due to the highly heterogeneity of these samples. A way to overcome this bottleneck is to use a single cell genomics approach that has been recently developed and allows the genome analysis of individual community members [21, 22].

Furthermore, powerful high-throughput tools can be provided with the use of phylogenetic oligonucleotide and functional gene arrays [23–25]. The so-called phylogenetic oligonucleotide arrays (POAs-PhyloChip) use a short oligonucleotide design against a phylogenetic marker gene (such as the 16S rRNA gene). They target polymerase chain reaction (PCR) amplified rRNA gene fragments, or directly retrieved community rRNA (genes) and can, at least in principle, be designed to detect any microorganism [26]. In contrast, functional gene arrays (FGAs) detect selected genes or gene families that encode key enzymes that are diagnostic for a certain metabolic pathway [27–29]. Therefore, these arrays are confined to diversity analysis of selected microbial guilds, while PhyloChips are best suited for detecting changes in the taxonomic composition of microbial populations.

In this review, we present a synopsis on (a) the functional gene arrays and phylogenetic oligonucleotide arrays that are currently available to the scientific community, (b) the next generation sequencing technologies with an emphasis on 16S rRNA amplicon pyrosequencing and, (c) single cell genomics, a new approach to study the microbial dark matter.

## 2. PhyloChip and Functional Gene Arrays

DNA microarrays are able to detect microbial sequences from any sample in a parallel and very fast, high throughput way. This technology has found applications across most sectors of life sciences, including environmental microbiology and microbial ecology [30]. Microbial ecological microarrays have been developed for investigating the composition and functions of microbial communities in environmental niches [31]. This tool is valuable in bacterial diversity studies since a single array can contain thousands of DNA sequences with a high degree of specificity [32].

The small subunit ribosomal RNA gene (16S rRNA) is the biomarker of choice for characterizing complex microbial communities [12, 33]. This biomarker is extensively used for phylogenetic analysis since it contains highly conserved and variable regions that allow a reliable and detailed microbial classification. The most comprehensive POA is the PhyloChip, which uses the Affymetrix format (Santa Clara, CA) [34–36]. The PhyloChip is updated regularly, and currently two versions of the DNA microarray are available. The development of the second generation PhyloChip (G2) started in 2002 and it became available in 2006. The PhyloChip G3 is currently available through the Second Genome Inc. PhyloChip G2 contains 297,851 perfect match (PM) and mismatch (MM) 16S rRNA gene probes for the detection of 842 subfamilies or 8,741 taxa, covering 121 bacterial and archaeal orders [37]. The remaining 209,093 probes are control probes or pathogen detection probes (Table 1) [25, 34, 38].

The design of PhyloChip G2 was based on over 30,000 16S rRNA gene sequences retrieved from the “Greengenes” database in March 2002 [26]. Chimeric sequences were filtered out and, subsequently, they were aligned resulting in 8,935 clusters (OTUs), which contained approximately 0–3% sequence divergence. The region selected for probe design was flanked on both sides by universally conserved segments that are used as PCR priming sites. These sites can be used to amplify bacterial and/or archaeal genomic materials. The probe selection strategy was to obtain an effective set of probes capable of correctly categorizing mixed amplicons into their proper OTUs. To correctly identify each OTU, a set of 11 or more specific 25-mers (probes) was designed. These probes were prevalent in members of a given OTU but dissimilar from sequences outside the given OTU. Probes that were complementary to target sequences were selected and termed perfect match (PM) probes. Each PM probe was paired with a control 25-mer termed mismatching (MM) probe, identical in all positions except the 13th base. The mismatching probe did not contain a central 17-mer complementary to sequences in any OTU. The target probe and MM probe constitute a probe pair analyzed together. The PhyloChip G2 is arranged in a grid of 732 columns and rows allowing the placement of 506,944 features. For the design of PhyloChip G3, a similar approach was adapted [23]. The Greengenes database was used and the filtered rRNA gene sequences were clustered to enable selection of perfectly complementary probes representing each sequence of a cluster. Amplicons that were containing 17-mers with sequence identity to a cluster were positioned in that cluster. This analysis gave rise to 59,959 clusters, each capturing an average of 0.5% sequence divergence (Table 1). These clusters were considered as operational taxonomic units (OTUs). The PhyloChip G3 provides an analysis spanning in 2 domains, 147 phyla, 1,123 classes, 1,219 orders, and 10,993 subfamilies [23].

One of the main advantages that the PhyloChip provides is the great sensitivitythat it delivers. A typical 16S PCR reaction with a yield of approximately 500 ng of amplicons provides more than 600 billion sequences, which allows even the less abundant populations to be tracked in addition to the dominant ones. Also, the PhyloChip has been shown to reveal greater diversity within a community when compared with rRNA Sanger sequencing clone libraries due to the placement of the entire gene product on the microarray compared with the analysis of up to thousands of individual molecules by traditional sequencing methods [39]. The main disadvantage of this technology is that the design of the PhyloChip, and in general of the DNA microarrays, does not allow the discovery and the characterization of novel taxa. This is true for the functional gene arrays as well since novel functions cannot be identified through this approach.

The PhyloChips G2 and G3 have been shown to provide identification resolution at the family to subfamily levels [34, 37, 40], and they have been used in over 80 publications. This technology has been used to successfully describe the microbial profile in a vast spectrum of complex ecosystems like solar salterns, industrial waste, olive-mill waste marine environments, coral reefs, air craft particulate air, soil, plant tissues, and various human microbiota [23, 25, 35, 36, 39, 41–44]. 

Utilization of the PhyloChip in olive-mill waste revealed a cultivar-dependent microbial profile [36]. With the implementation of the PhyloChip, a broader diversity was identified dominated by members of all classes of *Proteobacteria*, *Firmicutes*, *Bacteroidetes*, *Chloroflexi*, *Cyanobacteria* and *Actinobacteria*, while members of the phyla *Acidobacteria*, *Planctomycetes*, *Gemmatimonadetes*, and *Verrucomicrobia* and the candidate divisions OP3 (*Omnitrophica* [45]), TM7, AD3, marine group A (*Marinimicrobia* [45]), and SPAM were minor constituents of the bacterial biota.

The PhyloChip was used to associate microbial communities in aerosols [34]. Samples were collected over a 17-week period in San Antonio and Austin. Both sites were a part of a biosurveillance effort to detect bioterrorism threads. A diverse group of microorganisms associated with aerosol was detected with the PhyloChip. Sequences similar to or related to potential pathogens including *Campylobacteraceae*, *Helicobacteraceae*, and *Francisella*-like and bacteria related to *Bacillus anthracis*, *Rickettsia*, and *Clostridium* were identified.

Functional Gene Arrays (FGAs) are a special type of DNA microarrays containing probes for key genes involved in microbial functional processes. FGAs are composed of probes for key genes, involved in microbial functional processes of interest [29, 46, 47]. This type of array allows the simultaneous examination of many functional genes unlike PCR-based techniques that limit the number of genes that can be examined at one time [29, 46–48]. FGAs are especially useful for the study of environmental samples since the precise functions are not known due to the lack of cultured microorganisms and the high degree of diversity and metabolic flexibility that exists in microbial communities [49].

GeoChip 3.0 is the most comprehensive DNA microarray currently available for studying microbial communities associated with biogeochemical cycling, global climate change, bioenergy, agriculture, land use, ecosystem management, environmental cleanup and restoration, bioreactor systems, and human health. The design of GeoChip 3.0 involved the use of 56,990 gene sequences from 292 functional genes utilizing 27,812 probes. Eight degenerate probes for the 16S rRNA gene were used for positive controls, while 672 unique probes designed from hypothetical genes of seven sequenced genomes of hyperthermophiles were used as negative controls [50]. In GeoChip 3.0, 292 key enzymes/genes were used to target a variety of microbial mediated processes. In brief, a total of 41 enzymes/genes are selected to detect different functional processes of the carbon cycle, and 16 enzymes/genes are targeting the nitrogen cycling processes. Four enzymes/genes are used to detect the sulfur cycling of microbial communities, while three enzymes were targeting the phosphorus cycling of microbial communities. The enzymes hydrogenase and cytochrome detect energy metabolism processes of microbial communities, while a total of 41 genes/enzymes cover resistance mechanisms for Ag, Al, As, Cd, Co, Cr, Cu, Hg, Ni, Pb, Se, Te and Zn [49].

In GeoChip, the ability to monitor degradation pathways is also incorporated with a total of 173 genes/enzymes being used to detect and monitor the degradation of 86 organic contaminants commonly found in the environment [24, 51]. Finally, eleven genes for antibiotic resistance were also included. 

Based on GeoChip 3.0, the latest generation, GeoChip 4.0 in the NimbleGen format has been developed, which not only contains functional categories from GeoChip 3.0 but also includes additional functional categories, such as genes involved in stress responses, bacterial phages, and virulence [50, 52].

It can be postulated that future FGAs will be more comprehensive for the survey of diverse microbial communities, and at the same time they will be more specific for the detection and identification of microbial communities for particular ecosystems or functional processes of interest [50].

The microbial communities of a Gulf of Mexico coastal salt marsh were recently examined during and after the influx of petroleum hydrocarbons following the Deepwater Horizon oil spill using PhyloChip and GeoChip microarray analyses [53]. The abundance of phyla containing previously described hydrocarbon-degrading bacteria like *Proteobacteria*, *Bacteroidetes*, and *Actinobacteria*, increased in hydrocarbon-contaminated sediments and decreased once the hydrocarbon was below detection. Interestingly, the functional genes involved in the hydrocarbon degradation were enriched in hydrocarbon-contaminated sediments. Once the hydrocarbon concentration was reduced, the detection of functional genes involved in degrading alkanes, cycloalkanes, aromatic carboxylic acids, chlorinated aromatics, polycyclic aromatics, and other aromatics decreased significantly. 

In conclusion, DNA microarrays, that use multiple rRNAs or other phylogenetic markers, can be deployed to track variations in population structure and community function over time and space. The use of DNA microarrays based on selected genes (and gene variants) that are involved in interesting processes can be used to assess a community's ability to perform a collective function, such as biodegradation of contaminants, and monitor, for example, during bioremediation changes over relevant periods.

## 3. Next Generation Sequencing Technologies

The beginning of the modern DNA sequencing era began with the completion of the first draft of the human genome [54–56]. This was the turning point that led to further innovation and improved development of new advanced strategies of high-throughput DNA sequencing. These technologies are called the next generation sequencing (NGS), and this is the term that we are going to use throughout this review. The main principle in NGS involves DNA molecules that are being sequenced in a massively parallel fashion in a flow cell. The sequencing is conducted either in a continuous real-time manner or in a stepwise iterative process. During this highly parallel process, each clonal template or single molecule is independently sequenced and can be counted among the total sequences generated [57].

 At the moment, six platforms from the second and the third generation sequencing technologies are available with most platforms requiring short template DNAs (200–1000 bp) and with each template containing a forward and reverse primer binding site [58].

The GS FLX Pyrosequencer utilizes next generation sequencing technology known as pyrosequencing. The technique was first developed by Pal Nyren and his student Mostafa Ronhaghi at the Royal Institute of Technology in 1996 [59, 60] and is now available through Roche 454 Life Technologies. Pyrosequencing uses beads and starts with a single template molecule, which is amplified with emulsion PCR (emPCR). Millions of beads after the emPCR are loaded onto a picolitre plate, which is specially designed so that each well can hold only a single bead. The beads are sequenced in a parallel way by flowing pyrosequencing reagents across the plate. During pyrosequencing, the DNA is synthesized under a complex reaction that includes ATP sulfurylase, luciferase enzymes, adenosine 5′ phosphosulfate and luciferin substrates. These are incorporated in such a way that the pyrophosphate group releases upon addition of a nucleotide that results in the production of detectable light [61, 62]. The amount of light produced with pyrosequencing is proportional to the number of nucleotides incorporated. The FLX instrument provides 100 flows of each nucleotide during an 8 h run. This produces an average read length of 250 nucleotides. Analysis software examines the raw reads using various quality filters for removing poor quality sequences and mixed sequences that contain more than one initial DNA fragment per bead. Sequences that do not contain the initiating TCGA sequence are removed through the quality control test. The filtered reads yield approximately 100 Mb of quality data. It is accepted that FLX reads are of adequate length to assemble small genomes such as bacterial and viral genomes to high quality and contiguity [63]. For the specifications of the 454 technology please see Table 2.

Solexa developed the second commercial NGS platform. Solexa was subsequently acquired by Illumina and is now known by the name Illumina. Roche/454 and Illumina engage the principle of sequencing by synthesis. Illumina uses a solid glass surface that is very similar to a microscope slide, for capturing individual molecules and bridge PCR to amplify DNA into small clusters of identical molecules. These clusters at the end are sequenced with a strategy that is equivalent to Sanger sequencing. The difference lies in the use of dye-labelled terminators. 3′-O-Fluorophore-labeled nucleotides are used as reversible terminators of DNA polymerization. This reversible terminator ensures that, in one step, only one nucleotide can be incorporated. After the template is flooded with nucleotides and the binding step is accomplished, the unincorporated reagents are washed away and another round of dye-labelled terminators are added [64]. When compared with 454 sequencing, the Illumina sequencing technology achieves (a) much higher throughput (~1.5 Gbp/run) at the cost of significantly smaller read lengths and (b) high accuracy with error rates of less than 1% (Table 2). The sequencing approach is not affected by homopolymer runs to the same extent as the 454 technology [65]. 

The third commercial NGS technology was developed by SOLiD, and it is using ligation to determine sequences. Until recently, SOLiD was producing more reads than Illumina. Read lengths for SOLiD are user defined and range between 25 and 35 bp, while each sequencing run yields between 2 and 4 Gb of DNA sequence data [66, 67].

Ion Torrent uses a sequencing strategy similar to the 454, except that (i) hydrogen ions (H^+^) are detected (instead of a pyrophosphatase cascade) and (ii) sequencing chips conform to common design and manufacturing standards used for commercial microchips [68]. No cameras, lasers or fluorescent dyes are required with the Ion Torrent technology, while the common microchip design standards means that low-cost manufacturing can be used. Ion Torrent was purchased by Life Technologies in 2010. The first early instruments, the Ion Personal Genome Machine (PGM), was deployed in late 2010, while in September 2012, the Ion Proton was launched which is capable of producing larger outputs. Field effect transistors (FETs) are used to measure a change in pH in a microwell structure. To increase the throughput, the Ion Torrent sequencing chip makes use of a highly dense microwell array in which each well acts as an individual DNA polymerization reaction chamber. These chambers contain a DNA polymerase and a sequencing fragment. Below this layer of microwells an ion-sensitive layer is present, followed by a sublayer composed of a highly dense FET array aligned with the microwell array. Sequential cycling of the four nucleotides into the microwells enables primary sequence resolution since the FET detector senses the change in pH created during nucleotide incorporation and converts this signal to a recordable voltage change. While this method of ion sensing-based sequencing by synthesis offers great potential to reduce the cost of sequencing, there are several limitations with regards to sequencing complete genomes. The Ion PGM was mainly targeting small genomes given the output capability of the instrument (currently up to 1 Gb). The newly launched Ion Proton uses larger chips with higher densities and is said to be able to generate 10 Gb per run (Table 2). These characteristics make the technology suitable for exome and whole genome sequencing. Currently, the short read lengths place a burden on the reassembly process and limit the assembly of *de novo* sequencing projects due to an inability to read through long repetitive regions in the genome. Finally, error accumulation can occur if reaction wells are not properly purged between reaction steps, with an error rate of 1.78% being reported for Ion Torrent [63, 69].

The first commercial single-molecule sequencer (3rd generation) has been developed by Helicos. The high cost of the instruments and short read lengths unfortunately limited adoption of this platform, and at the moment Helicos no longer sells instruments; instead it conducts sequencing via a service centre model. 

PacBio has developed an instrument that sequences individual DNA molecules in real time [70]. Individual DNA polymerases are attached to the bottom of 50 nm wide wells that are termed zero-mode waveguides (ZMWs). Each polymerase is allowed to carry out second strand DNA synthesis in the presence of *γ*-phosphate fluorescently-labeled nucleotides. The width of the ZMW is such that light cannot proliferate through the waveguide, but energy can penetrate a short distance and excite the fluorophores attached to those nucleotides that are in the vicinity of the polymerase at the bottom of the well. As each base is incorporated, a distinctive pulse of fluorescence is detected in real time. The first instruments were deployed in late 2010. The low cost per experiment and fast run times have generated much enthusiasm for this platform, especially among investors. Although high accuracy can be achieved through circular consensus sequencing, which involves sequencing shorter templates multiple times, this instrument generates single-pass reads that average less than 85% nucleotide accuracy [69].

For each technology, there is a trade-off between advantages and disadvantages. The 454 technology delivers the longest read length but with the lowest throughput (8 MB/h during a 9 h run—Table 2) and suffers from errors in homopolymeric tracts, even when assembles are at high coverage. MiSeq (Illumina) generates the highest throughput per run and lowest error rate of the instruments but delivers shorter read lengths than those of the 454. Ion Torrent currently produces short reads and the worst performance with homopolymers, although the new chemistry has improved performance. Ion Torrent delivers the fastest throughput (80–100 Mb/h—Table 2) and shortest run time of an approx. 3 h. This platform has also shown the greatest improvement in performance in recent months [69, 71].

## 4. 16S rRNA Pyrosequencing and Hypervariable Regions 

Using the 16S ribosomal RNA gene as a phylogenetic marker was a real breakthrough for microbial ecology studies, with several culture-independent methods being developed since Pace et al. [72] proposed the direct cloning of environmental DNA. PCR-based molecular techniques enabled the description of microbial taxonomic diversity: (a) by means of fingerprinting methods, which separate rDNA fragments according to their length and/or nucleotide composition like denaturing/temperature gradient gel electrophoresis (DGGE/TGGE) [73], restriction fragment length polymorphisms (RFLP) [74], terminal restriction fragment length polymorphism (T-RFLP) [75], single-strand conformation polymorphism (SSCP) [76], and automated rRNA intergenic spacer analysis (ARISA); (b) by microscopy using FISH (fluorescence *in situ* hybridization) and derived methods (CARD-FISH, MAR-FISH); and (c) by cloning 16S rRNA gene fragments and subsequently sequencing the clones following the Sanger sequencing method. It is true that fingerprinting technologies enable the processing of many samples, but they are inadequate for taxonomic identification and suffer from a lack of resolution. Finally, cloning/sequencing and FISH are not compatible with high-throughput approaches.

Cloning and sequencing of the 16S ribosomal RNA gene using conserved broad-range PCR primers was and still is the most common molecular approach for estimating the microbial diversity. But, with the development of NGS technologies, direct sequencing of PCR amplicons became feasible [77, 78]. It is true that the rapid development of sequencing technologies has opened a new dimension in biodiversity analysis, but the most critical step for an accurate rDNA amplicon analysis remains the correct choice of primers and the hypervariable regions that will be targeted [78, 79].

**Figure 1:**
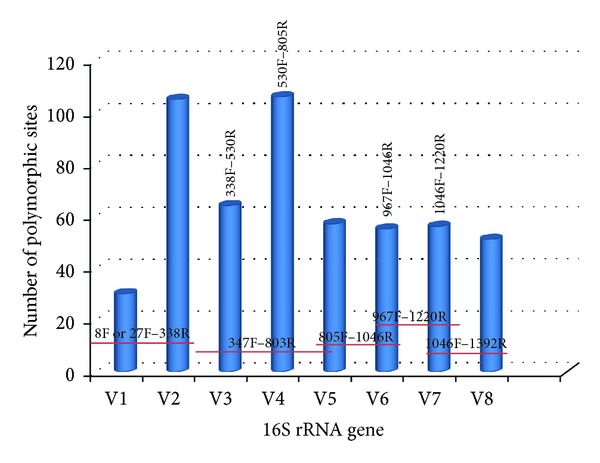
Graphical presentation of the variable regions within the 16S rRNA gene and location of corresponding primer pairs that can be deployed for specific region amplification. Variable regions presented exclude poorly supported areas, and for this reason the V9 region is not presented.

The 16S rRNA gene in bacteria is comprised of interspersed conserved and variable sequences including eight (8) hypervariable regions (V1–V8) (Figure 1). The eight hypervariable regions spanned nucleotides 69–99, 137–242, 433–497, 576–682, 822–879, 986–1043, 1117–1173, and 1243–1294 for V1 through V8, respectively [23, 80]. These hypervariable regions range in size from approximately 50 to 100 bases in length, while sequences differ with respect to variation and corresponding utility for universal microbial identification (Figure 1). Hypervariable regions of the 16S rRNA gene are flanked by conserved sequences (Figure 1) and this enables the design of “universal” PCR primers that can amplify 16S rRNA hypervariable regions from a large number of different bacteria species [39, 80, 81] (see Table 3).

The most critical step for accurate characterization of bacterial and archaeal communities using rDNA amplicon analysis is the choice of primers. Using suboptimal primers pairs will lead to underrepresentation or underselection against single species or even whole groups which can lead to questionable biological conclusions [40]. Different hypervariable regions evolve at different rates, and different species of the same genus may be similar in some hypervariable regions and more divergent in others [82]. Primer bias occurs when the selected primers do not anneal to the DNA from all members of the community equally, but preferentially amplify certain taxonomic groups [6]. This can lead to the failure in detecting some bacterial/archaeal species since in rare biospheres bacteria and archaea can never be identified if the employed primers are not applicable to them. This will lead to incomplete surveys in metagenomic studies [83].

It has been shown that sequences of 500–700 bp are required for phylogenetic discrimination at the species levels [84, 85]. With the NGS technologies, fragments of up to 700 bp are being sequenced regularly with investigations supporting that use of the V1, V2, and V3 regions for deep sequencing and characterization of bacterial and archaeal sequences [86, 87]. Others suggest that regions generated using primer pairs 8F-338R and 967F-1046R for V6 overestimate species richness and promote the V4–V6 generated using primer pairs 530F-805R, 805F-1046R and 967F-1220R as the most appropriate [82, 88] (Table 3). Also, fragments encompassing the V3, V7, and V7+V8 hypervariable regions (generated using primer pairs 338F-530R, 1046F-1220R, and 1046F-1392R) underestimated species richness [82] (Table 3). Recent studies demonstrated that the V7-V8 fragments achieve better microbial community coverage from a complex ecosystem [88]. It is highly recommended to use primers that are targeting two regions of the 16S rRNA gene in all deep-sequencing efforts when trying to characterize highly heterogeneous microbial communities [81, 88], although good representation of a microbial community has been achieved by targeting single hypervariable regions [89].

## 5. NGS Amplicon Sequencing in Microbial Ecology

In the recent years, mass sequencing of environmental samples has been the leading approach for microbial ecology studies. Irrespective of the ecosystem studied, the vast majority of the studies deployed the 454 pyrosequencing platform, although Illumina-based studies are in the increase.

Soil bacterial diversity was examined using NGS technologies, and this approach revealed that the agricultural management of the soil can influence the diversity of bacteria and archaea [90–92]. The pH is the principal diversity driver for both *Bacteria* and *Archaea* [92–94] with the *Archaea* being highly correlated only with pH. The fungal community composition was less strongly affected by pH [93]. Soil fungal diversity was the focus of other studies in forest and agricultural ecosystems. Analysis using ITS amplicons revealed that in forests most species belong to the Dikarya subkingdom (Ascomycota and Basidiomycota), with the Agaricomycetes being the most dominant fungal class [95]. In agricultural ecosystems, it has been revealed that the diversity of the fungal community declines with soil depth with communities forming distinct groups among the strata [96]. 

Marine environments have been used in studies with NGS technologies. Analysis of the 18S rRNA gene identified members from all six eukaryotic supergroups. It also revealed that the eukaryotic microbiota was dominated by dinoflagellates and close relatives which demonstrates the importance of this group to marine ecosystems [97]. The use of 18S rDNA pyrosequencing enabled the characterization of uncultured eukaryotes like flagellates, which are known as MArine STramenopiles (MAST) [98]. Finally, the use of 16S rRNA tag pyrosequencing analysis from a temperate marine coastal site over a period of 6 years, suggested that seasonal changes in environmental variables are more important than trophic interactions [99]. 

NGS technologies have been applied in freshwater environmental samples. Monchy et al. [100] used cloning/sequencing and SSU tag pyrosequencing to study the fungal diversity in freshwater lake ecosystems. This study indicated that geographical, physical, and chemical factors of the biotope influence the species community structure and spatial variability. In a very interesting study by Logares et al. [101], the bacterioplankton communities in a unique system of coastal Antarctic lakes exposed to progressive long-term environmental change was examined using 454 pyrosequencing of the 16S rDNA gene (V3-V4 regions). Progressive long-term salinity change appears to have promoted the diversification of bacterioplankton communities by modifying the composition of ancestral communities and by allowing the establishment of new taxa [101]. NGS technologies are more robust in describing the structuring of understudied or highly divergent populations. For instance, new putative clades belonging to Mamiellophyceae, Foraminifera, Dictyochophyceae, and Euglenida were recently detected in eight freshwater ecosystems using rDNA pyrotag data [102]. 

Air microbial diversity has been recently studied using pyrosequencing technologies in the New York City subway platforms and associated sites [103]. Eukaryotic diversity was mainly fungal, dominated by organisms of types associated with wood rot. Bacterial diversity was dominated by human skin bacterial species including *Staphylococcus epidermidis* (the most abundant and prevalent commensal of the human integument), *S. hominis*, *S. cohnii*, *S. caprae*, and *S. haemolyticus*, while no organisms of public health concern were identified [103].

## 6. Single Cell Genomics

Recent estimates predict that the number of microbial species in the world are well into millions, and based on the rRNA phylogeny, these species fall within approximately 60 major lines of descent within the bacterial and archaeal domains [33, 104]. From the 60 major lines, at least half have no cultivated representatives, and they are called “candidate” phyla. Even from the phyla with culturable representatives, 88% belong to only four bacterial phyla, the *Proteobacteria*, *Firmicutes*, *Actinobacteria*, and *Bacteroidetes*. One approach for sequencing candidate phyla is by deploying a metagenomic approach, thus obtaining genome sequences from the microbial dark mater through direct sequencing of DNA from microbial communities [3]. The use of such approach enabled the draft to complete genome recovery from candidate divisions: (a) WWE1/*Cloacimonetes* (Wastewater Evry 1) with the *Cloacamonas acidaminovorans*, (b) NC10 with the *Methylomirabilis oxyfera*, (c) OP1/*Acetothermia* with the *Acetothermum autotrophicum, *and (d) from Korarchaeota the *Korachaeum cryptofilum* [105–108]. With the development of new bioinformatic tools in combination with deep metagenomic sequencing low abundant genomes have been recovered including members of candidate phyla like OP11 (*Microgenomates*), OD1 (*Parcubacteria*), and GNO2 (*Gracilibacteria*) [109].

**Figure 2:**
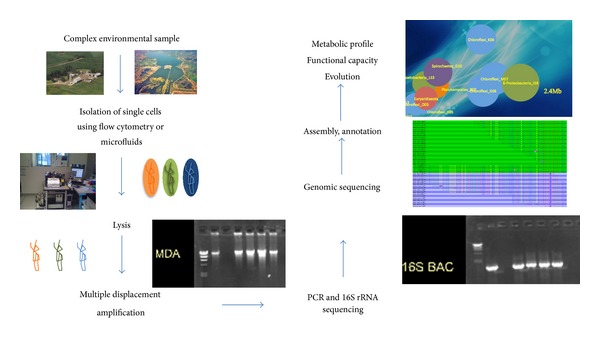
General overview of the single cell genomics approach.

Another approach that can be deployed in order to obtain genomic data from candidate divisions is the single cell genomics technique. With this approach, cells from any environmental sample can be isolated, and after amplification the DNA can be sequenced [110]. In more detail, almost any environmental sample can be processed immediately or stored in the presence of betaine or glycerol so that the integrity of the cells be preserved [111]. The next step involves cell separation and this is currently achieved with the use of fluorescence-activated cell sorting (FACS) [111–113]. In comparison to micromanipulation, FACS minimizes the risk of contamination since a few picoliters of sample are sorted each time. Cell lysis follows (Figure 2) with the most effective method being the alkaline lysis [114]. Whole Genome Amplification can be achieved through the Multiple Displacement Amplification (MDA) approach producing long, overlapping amplicons that can be used with the NGS technologies. *In silico* DNA normalization and specialized software have been developed to counteract the drawbacks of MDA [111, 115]. Before the genome sequencing of the Single Amplified Genomes (SAGs) using NGS technologies, a PCR step can be included for screening purposes. Amplification and Sanger sequencing of the 16S rRNA enables phylogenetic characterization of the SAGs (Figure 2). The recovery of genomic information from single cells varies from 0% up to complete genomes and depends on the intrinsic properties of the cell and on the components of the SCG pipeline deployed. For instance, the above described approach has been successful to target members of the candidate phyla TM7, OP11 (*Microgenomates*) and *Poribacteria* [116–118] and led to the recovery of genomic sequences of microorganisms from several deep-branching phylogenetic groups with no cultured representatives. Other examples include *Picobiliphytes* and divergent groups of aquatic *Proteobacteria*, *Flavobacteria,* and *Archaea* [111, 112, 119–122].

With the single cell genomics (SCG) approach, for, first time, a direct link between phylogenetic and metabolic markers of uncultured bacteria and archaea is possible. A recent example is the discovery of chemolithoautotrophical pathways in uncultured *Proteobacteria* [111], which reconciliate the current discrepancies in dark ocean's carbon budget. SCG is also capable of producing reference genomes of the uncultured microorganisms, enabling the study of complex ecosystems. For instance, Single Cell Genomics have been deployed to investigate biogeographic distribution of uncultured marine *Flavobacteria*, and marine bacterioplankton that were involved in the degradation of hydrocarbons during the Deepwater Horizon oil spill [112, 123]. 

In a recent study by Rinke et al. [45], an SCG approach was deployed targeting 201 uncultivated archaeal and bacterial cells that belong to 29 major mostly uncharted branches of the tree of life, the so-called “microbial dark matter.” Sequencing of 201 single amplified genomes enabled the resolution of many intra- and interphylum-level relationships and enabled the characterization of new superphyla. The first one, *Terrabacteria*, comprises terrestrial bacterial phyla of *Actinobacteria*, *Cyanobacteria*, *Thermi* (*Deinococcus-Thermus*), *Chloroflexi*, *Firmicutes,* and *Armatimonadetes*. This superphylum comprises monoderm (single membrane) and atypical lineages. The second superphylum is the *Patescribacteria* (patesco (Latin), meaning bare), which reflects the reduced metabolic capacities of these lineages. Finally, the superphylum DPANN was identified composed by the *Diapherotrites* (pMC2A384), *Parvarchaeota*, *Aenigmarchaeota* (DSEG), and *Nanohaloarchaeota*. Finally, substantive genomic data for 11 bacterial candidate divisions and several highly divergent archaeal groups related to *Nanoarchaeota* were resolved as monophyletic groups. This enabled the proposition of names for these candidate divisions based on their inferred physiology and distinguishing properties (Table 4).

SCG generates a whole new way for exploiting bacterial diversity since, to date, biotechnological applications rely almost exclusively on the part of the microbial world that can be cultured, that is, less than 1% of the microbial diversity. Although metagenomic-based bioprospecting provides an alternative [124–126], the main advantage that the single cell genomics approach offers is that rather than individual genes of the uncultured microorganisms the whole genome is sequenced. This approach enables the construction of complex metabolic pathways, ensuring that all discovered genes are originating from the same cell. Some early examples of biotechnological exploitations through the use of the single cell genomic technique include recoveries of polyketide biosynthesis pathways from sponge symbionts [118, 127] and the discovery of uncultured microorganisms that degrade specific macromolecules and fix CO_2_ through chemoautotrophy [111, 128, 129].

## 7. Conclusion

The study of the microbial diversity is important for understanding the link between diversity, community structure, and function. The most advanced technologies in this quest are DNA microarrays, NGS, and single cell genomics. DNA microarrays provide a fast and high-throughput approach for the parallel detection of microbes from any sample. The most comprehensive phylogenetic oligonucleotide array is the PhyloChip, which uses the Affymetrix format with a current analysis of 59,959 OTUs. GeoChip 3.0 is an advanced Functional Gene Array with a resolution of 292 key enzymes/genes containing more than 28,000 probes. These DNA microarrays revolutionized the way that complex microbial communities are being studied. The development of the new-generation sequencing technologies challenged the use of DNA microarrays in microbial community studies. It appears that in low to medium complexity ecosystems use of the NGS technologies is the most promising approach. In highly complex ecosystems, the sequence-based technologies suffer from random sampling, under sampling, and rRNA interference [130–132]. DNA microarrays like PhyloChip and GeoChip are excellent tools when highly complex ecosystems are examined due to their unique features like (a) rapid output, (b) quick sample preparation, (c) community comparison, (d) quick data analysis, and (d) resistance to contaminants. 

NGS technologies will continue to improve both accuracy and throughput with benchtop sequencers becoming the standard equipment in individual labs. We believe that amplicon analysis will become in the near future a quick screening technique, preliminary to more detailed metagenomic studies, rather than the final stage in ecological analysis.

SCG provides the ability to read genetic information at the basic level of biological organization. The capacity to sequence any genomic region of an uncultured cell provides for the first time a direct link between phylogenetic and metabolic markers. The power of SCG has been demonstrated by revealing metabolic features and *in situ* interactions that have not been able to be characterized before with any other molecular approach. SCG offers a unique opportunity to obtain genomic information from major uncultivated microbial lineages.

Finally, we believe that new approaches exploiting NGS technologies and Single Cell Genomics jointly will be developed targeting genomes and associating them with quantitative measurements. This approach can target all active members (abundant and rare) of a given ecosystem, measure the transcribed genes, and obtain the full genome. 

## Figures and Tables

**Table 1:** Comparison of the main phylogenetic oligonucleotide and functional gene arrays.

Gene array	Probe type	No. of probes	Analysis provided
PhyloChip G2	25-mer oligos	297,851	8,935 OTUs
PhyloChip G3	25-mer oligos	1,100,000	59,959 OTUs
GeoChip 3.0	50-mer oligos	27,812	292 gene families
GeoChip 4.0	50-mer oligos	120,054	539 gene families^a^

^a^GeoChip contains genes targeting human microbiomes in 139 functional gene families with 36,062 probes.

**Table 2:** Technical specifications of Next Generation Sequencing platforms.

Platform	454	Illumina	Life technologies ABI/SOLID	Helicos biosciences heliscope	Ion torrent	Pacific biosciences
Year of availability	2005	2006	2006	2007	2010	2010
Sequencing length	200–700 bp	Up to 150 bp	35–50 bp	25–55 bp	~200 bp	1500 bp
Sequence yield per run	700 Mb	2–600 Gb^a^	120 Gb	35 Gb	20–50 Mb on 314 chip 100–200 Mb on 316 chip Gb on 318 chip	100 Mb
Run time	23 h	27 h–11 days	7-8 days	3–6 days	2 h	2 h
Technology	emPCR, pyrosequencing	Polonies, cleavable dye terminators	emPCR, ligation with cleavable dye terminators	True Single Molecule Sequencing (tSMS)Single base, reversible dye terminator extension reactions	emPCR, H+ detection	Single Molecule Real Time (SMRT) sequencing dyes that are phospholinked to the nucleotide, very sensitive fluorescent detection in zero mode waveguides

^a^2 Gb for the MiSeq and 600 Gb for the HiSeq2000.

**Table 3:** Oligonucleotide primers that can be used for 16S rRNA variable region PCR amplification and sequencing of bacterial 16S rRNA genes.

Primer	Sequence 5′ to 3′	Reference
8F	AGAGTTTGATCCTGGCTCAG	[133]
27F	AGAGTTTGATCMTGGCTCAG	[134]
338R	GCTGCCTCCCGTAGGAGT	[135]
338F	ACTCCTACGGGAGGCAGC	[136, 137]
530R	AATACGGAGGGTGCAAGCGT	[136, 137]
530F	ACGCTTGCACCCTCCGTATT	[136, 137]
805R	GGATTAGATACCCTGGTAGTC	[136, 137]
805F	GACTACCAGGGTATCTAATCC	[136, 138, 139]
967F	CAACGCGAAGAACCTTACC	[138, 139]
1046F	ACAGCCATGCAGCACCT	[138]
1046R	AGGTGCTGCATGGCTGT	[138, 139]
1220R	GTAGCRCGTGTGTMGCCC	[138, 139]
1392R	ACGGGCGGTGTGTRC	[134]

**Table 4:** Proposed names for candidate phyla and associated superphyla (adapted from Rinke et al. [45]).

Superphylum	Candidate phylum	Proposed name	Etymology
PVC	OP3	Omnitrophica	Omnitrophus, eating all Om.ni.tro'phi.ca. A higher taxonomic unit comprising the genus *Omnitrophus *

FCB	SAR406 (Marine Group A)	Marinimicrobia	Marinimicrobium, a marine microbe Ma.ri.ni.mi.cro'.bi.a. A higher taxonomic unit comprising the genus *Marinimicrobium *
	WS3	Latescibacteria	Latescibacter a hiding small rod La.tes.ci.bac.te'ri.a. A higher taxonomic unit comprising the genus *Latescibacter *
	WW1	Cloacimonetes	Cloacimonas a unit from a sewer Clo.a.ci.mo.ne'tes. A higher taxonomic unit comprising the genus *Cloacimonas*.

	OP8	Aminicenantes	Aminicenans a (bacterium) degrading amino acids A.mi.ni.ce.nan'tes. A higher taxonomic unit comprising the genus *Aminicenans*.

Patescibacteria	OP11	Microgenomates	Microgenomatus; an organism with a small genome size (~1 Mbp) A higher taxonomic unit comprising the genus *Microgenomatus *
	OD1	Parcubacteria	Paceibacter Pace's bacterium norman'i.i. N.L. gen. N. of Norman, referring to Norman Pace (Norman Richard Pace, Jr. is an American biochemist, Distinguished Professor of Molecular, Cellular and Developmental Biology at the University of Colorado, and principal investigator at the Pace lab) -parcus (lat.), thrifty A higher taxonomic unit comprising the genus *Paceibacter *
	GN02 (BD1-5)	Gracilibacteria	-gracilis (lat.), slim, slender, slight, meager, simple A higher taxonomic unit comprising the genus *Altimarinus *

	OP9	Atribacteria	A.tri.bac.te'ri.a. N.L. n. A higher taxonomic unit comprising the genus *Caldatribacterium *
	EM19	Calescamantes	Ca.lesc.a.man'tes. L. v. calesco, to become warm, grow hot; L. v. amo, to love, N.L. n. Calescamantes heat lovers A higher taxonomic unit comprising the genus *Calescibacterium*
	CD12 (BHI80-139)	Aerophobetes	A.er.o.pho'bus. Gr. n. aer, air; Gr. adj. phobos, fear. N.L. n. Aerophobus, fearing of air (i.e., oxygen). A higher taxonomic unit comprising the genus *Aerophobus *
	NKB19	Hydrogenedentes	Hydrogenedens a hydrogen consumer te.re.phtha'li.cus. N.L. n. (acidum) terephthalicum, terephthalic acid. N.L. adj. terephthalicus, referring to the environment of isolation, a terephthalate reactor. A higher taxonomic unit comprising the genus *Hydrogenedens. *
	OP1	Acetothermia	Acetothermus indicates a vinegar organism living in hot places and ‘‘autotrophicum” (au.to.tro'phi.cum. Gr. pron.autos self; Gr. adj. trophikos nursing, tending or feeding; N.L. neut. adj. autotrophicum selfnursing or self-feeding). A.ce.to.ther'mi.a. A highe taxonomic unit comprising the genus *Acetothermum *
	Oct-Spa1-106	Fervidibacteria	Fer.vi.do.bac'ter. L. adj. fervidus, hot, steaming; -i-connecting vowel; N.L. n. bacter, a rod; N.L. n. Fervidibacter a hot rod. sac.cha'ri. N.L. n. saccharum, sugar; N.L. gen. n. sacchari, of sugar Fer.vi.di.bac.te'ri.a. A higher taxonomic unit comprising the genus *Fervidibacter *
DPANN	pMC2A384	Diapherotrites	(Dia.phe.ro.tri'tes. Gr. v. diaphero, to differ; Gr. adj. trítos the third; N.L. n. Diapherotrites the 3rd HSM group discovered) A higher taxonomic unit comprising the genus *Iainarchaeum *
	ARMAN group	Parvarchaeota	Parv.ar.chae'a. A higher taxonomic unit comprising the genera *Parvarchaeum* and *Micrarchaeum *
	DSEG	Aenigmarchaeota	Ae.nig.mar.chae'a. A higher taxonomic unit comprising the genus *Aenigmarchaeum *
		Nanohaloarchaeota	Na.no.ha.lo.ar.chae.o'ta. Gr. n. nanos, dwarf; Gr. n. hal, -los, salt; Gr. adj. archaios, old; N.L. suffix -ota ending to design a phylum; N.L. n. Nanohaloarchaeota, small salt-loving Archaeota. A higher taxonomic unit comprising the genus *Nanosalina *
